# Antifungal and Cytotoxic Activities of Sixty Commercially-Available Essential Oils

**DOI:** 10.3390/molecules23071549

**Published:** 2018-06-27

**Authors:** Chelsea N. Powers, Jessica L. Osier, Robert L. McFeeters, Carolyn Brianne Brazell, Emily L. Olsen, Debra M. Moriarity, Prabodh Satyal, William N. Setzer

**Affiliations:** 1 Department of Chemistry, University of Alabama in Huntsville, Huntsville, AL 35899, USA; cnp0007@uah.edu (C.N.P.); jlo0011@uah.edu (J.L.O.); rlm0004@uah.edu (R.L.M.); 2 Department of Biological Sciences, University of Alabama in Huntsville, Huntsville, AL 35899, USA; cbb0027@uah.edu (C.B.B.); elo0001@uah.edu (E.L.O.); moriard@uah.edu (D.M.M.); 3 Aromatic Plant Research Center, 615 St. George Square Court, Suite 300, Winston-Salem, NC 27103, USA; psatyal@aromaticplant.org

**Keywords:** *Aspergillus niger*, *Candida albicans*, *Cryptococcus neoformans*, cytotoxicity, human breast tumor

## Abstract

There is an urgent and unmet need for new antifungal therapies. Global fungal infection rates continue to rise and fungal infections pose increasing burdens on global healthcare systems. Exacerbating the situation, the available antifungal therapeutic arsenal is limited and development of new antifungals has been slow. Current antifungals are known for unwanted side effects including nephrotoxicity and hepatotoxicity. Thus, the need for new antifungals and new antifungal targets is urgent and growing. A collection of 60 commercially-available essential oils has been screened for antifungal activity against *Aspergillus niger*, *Candida albicans*, and *Cryptococcus neoformans*, as well as for cytotoxic activity against MCF-7 and MDA-MB-231 human breast tumor cell lines; the chemical compositions of the essential oils have been determined by gas chromatography-mass spectrometry (GC-MS). Ten essential oils showed remarkable antifungal and cytotoxic activities: Indian, Australian, and Hawaiian sandalwoods; melissa; lemongrass; cilantro; cassia; cinnamon; patchouli; and vetiver.

## 1. Introduction

Fungi are ubiquitous in nature. Of the estimated 1.5 million species of fungi [1], there are approximately 100 species that cause human infection [2]. These infections include aspergillosis, candidiasis, and cryptococcosis, among others [3]. Invasive fungal infections from these opportunistic pathogens have been increasing in recent decades, causing substantial morbidity and mortality [2,4]. The most common *Aspergillus* species causing pulmonary aspergillosis is *A. fumigatus*, but *A. flavus*, *A. terreus*, and *A. niger* can also cause *Aspergillus* lung disease, particularly in immunosuppressed individuals [5]. Likewise, the principle agents of candidiasis are *Candida albicans*, *C. glabrata*, *C. tropicalis*, *C. parapsilosis*, and *C. krusei* [6]. *Cryptococcus neoformans* is the main fungal species responsible for cryptococcosis, but *Cryptococcus* taxonomy has undergone several revisions [7]. Treatment options for invasive fungal infections include amphotericin B, as well as several azole compounds, such as fluconazole and itraconazole [8]. However, there have been severe side effects associated with these antifungal agents [8], and antifungal resistance continues to increase [9].

Essential oils are complex mixtures of volatile compounds derived principally from higher plants [10]. These materials have been used to treat human infections and other maladies for centuries. The biological activities associated with essential oils depend on the compositions, both the concentrations of the major components and the possible synergistic interactions with minor components. In this report, we present the antifungal screening of a collection of 60 essential oils obtained from commercial sources against *Aspergillus niger*, *Candida albicans*, and *Cryptococcus neoformans*. In addition, the essential oils were also screened against two human breast tumor cell lines, MCF-7 (estrogen receptor positive breast adenocarcinoma) and MDA-MB-231 (estrogen receptor negative breast adenocarcinoma).

## 2. Results

The antifungal and cytotoxicity screening results are summarized in Table 1. The most active essential oils, both in terms of antifungal activity and cytotoxic activity, were the sandalwood species (*Santalum album*, *S. austrocaledonicum*, and *S. paniculatum*), rich in santalols; cassia (*Cinnamomum cassia*) and cinnamon (*C. zeylanicum*), both dominated by cinnamaldehyde; lemongrass (*Cymbopogon flexuosus*), melissa (*Melissa officinalis*), and cilantro (*Coriandrum sativum* leaf oil), which were dominated by aldehydes; patchouli (*Pogostemon cablin*), rich in patchouli alcohol; and vetiver (*Vetiveria zizanoides*), with isovalencenol and khusimol as major components. Of the fungal species tested, *Cryptococcus neoformans* was the most susceptible and *Candida albicans* was the least sensitive. Both breast tumor cells lines showed similar activities and correlated well with *C. neoformans* antifungal activity.

**Figure 1:**
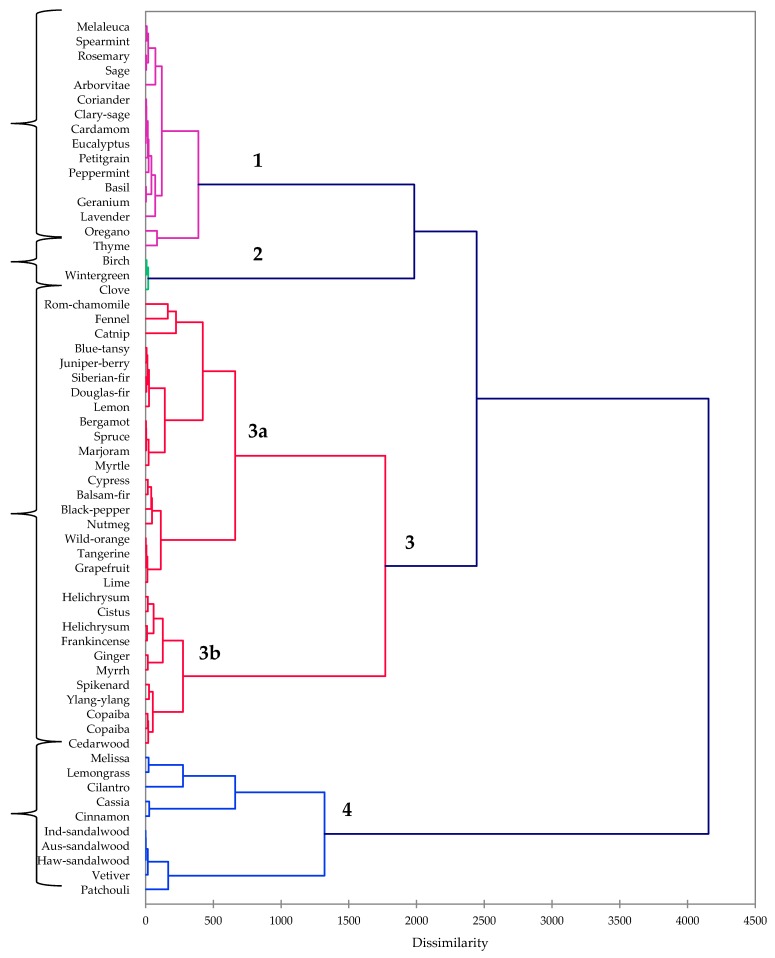
Dendrogram obtained from the agglomerative hierarchical cluster analysis of 60 essential oil compositions; antifungal and cytotoxic activities.

A hierarchical cluster analysis (Figure 1) revealed four apparent clusters based on compositions and bioactivities: (**1**) a largely inactive cluster that is dominated by oxygenated monoterpenoids; (**2**) an inactive cluster with aromatics as the predominant chemical class; (**3**) a largely inactive cluster, dominated by monoterpene and sesquiterpene hydrocarbons; and (**4**) the biologically active cluster, which is rich in oxygenated sesquiterpenoids and aldehydes.

## 3. Discussion

Cluster **1** is characterized as being composed largely of oxygenated monoterpenoids and is relatively inactive. Notable members of cluster **1** are *Melaleuca alternifolia*, *Salvia officinalis*, *Eucalyptus radiata*, *Origanum vulgare*, and *Thymus vulgaris*. Oxygenated monoterpenoids such as linalool, terpinen-4-ol, α-terpineol, borneol, camphor, or thujones are largely inactive against fungi, as well as tumor cells [11,12,13]. On the other hand, 1,8-cineole has shown moderate antifungal activity [11,14], and the activity of 1,8-cineole is likely responsible for the moderate antifungal activity of *Eucalyptus radiata* essential oil (minimum inhibitory concentrations (MIC) = 313 and 156 μg/mL against *A. niger* and *C. neoformans*, respectively). Tea tree (*Melaleuca alternifolia*) oil had previously shown only marginal antifungal activity, attributed to the active components terpinen-4-ol and and α-terpineol [15], and in this current work, we find only marginal antifungal activity (MIC = 625 μg/mL). In agreement with an earlier work [16], *Salvia officinalis* essential oil showed only marginal antifungal activity (MIC ≥ 625 μg/mL).

Interestingly, thyme (*Thymus vulgaris*; 43.9% thymol and 14.4% carvacrol) essential oil was not cytotoxic in this study. Oregano oil (*Origanum vulgare*; 74.2% carvacrol), on the other hand, was moderately cytotoxic (IC_50_ = 35.3 and 60.1 μg/mL on MCF-7 and MDA-MB-231 cells, respectively). Both thyme and oregano oils showed similar antifungal profiles with MIC = 156, 313, and 78 μg/mL against *A. niger*, *C. albicans*, and *C. neoformans*, respectively. The phenolic monoterpenoids, carvacrol and thymol, are likely responsible for the observed antifungal activities [17,18,19]. The biological activity of thyme essential oil depends on the thymol concentration; there are several chemotypes of thyme with vastly different concentrations of thymol [20].

Cluster **2** contained only three essential oils, all dominated by aromatic constituents: Birch (*Betula lenta*, 99.9% methyl salicylate), wintergreen (*Gualtheria fragrantissima*, 99.7% methyl salicylate), and clove (*Eugenia caryophyllata*, syn. *Syzygium aromaticum*, 80.6% eugenol and 10.5% eugenyl acetate). Neither birch nor wintergreen oils were antifungal or cytotoxic. However, clove oil was moderately antifungal (MIC = 156, 313, and 156 μg/mL against *A. niger*, *C. albicans*, and *C. neoformans*, respectively). Clove oil had previously demonstrated moderate antifungal activity against *A. niger* [21] and *C. albicans* [22], which can be attributed to the high concentration of eugenol [23].

Cluster **3** can be subdivided into a sub-cluster rich in monoterpene hydrocarbons (**3a**) and a sub-cluster with both monoterpene hydrocarbons and sesquiterpene hydrocarbons (**3b**). Sub-cluster **3a** is made up of gymnosperm essential oils and the *Citrus* essential oils and are, by and large, inactive.

Sub-cluster **3b**, on the other hand, has significant concentrations of sesquiterpenoids and generally showed moderate cytotoxic activity. Thus, for example, *Cistus ladanifer* essential oil had IC_50_ values of 36.6 and 46.3 μg/mL against MCF-7 and Hs578T cell lines; copaiba oils, rich in β-caryophyllene, showed moderate cytotoxic activities on both MCF-7 and MDA-MB-231 cells (IC_50_ values range from 22.7 to 67.2 μg/mL). Frankincense (*Boswellia carteri*) essential oil is also rich in β-caryophyllene and showed comparable cytotoxic activity. Sesquiterpene hydrocarbons, such as β-caryophyllene and α-humulene, have shown moderate cytotoxic activity against several human tumor cell lines [11,13,24]; the relatively high concentrations of sesquiterpene hydrocarbons in the essential oils of sub-cluster **3b** may account for the observed moderate cytotoxicities.

Cedarwood oil (the wood essential oil of *Juniperus virginiana*) had previously shown excellent cytotoxic activities against MCF-7 (IC_50_ = 3.99 μg/mL) and MDA-MB-231 (IC_50_ = 4.32 μg/mL) [25]. In our current study, however, *J. virginiana* wood oil was less active against these two cell lines (IC_50_ = 37.2 and 35.7 μg/mL, respectively), and showed only marginal antifungal activity (MIC = 625, 625, and 313 μg/mL against *A. niger*, *C. albicans*, *and C. neoformans*, respectively).

Cluster **4** is made up of the essential oils that showed both antifungal and cytotoxic activities. The sandalwood essential oils were particularly active against *C. neoformans* (MIC = 20 μg/mL) and MCF-7 cells (IC_50_ = 9.4, 9.5, and 13.3 μg/mL for *S. album*, *S. austrocaledonicum*, and *S. paniculatum*, respectively). Sandalwood oils were less effective against *A. niger* (MIC = 156–313 μg/mL) and only marginally active against *C. albicans* (MIC = 625 μg/mL), but still exhibited cytotoxic activity to MDA-MB-231 cells (IC_50_ = 19–24 μg/mL) and showed similar activities against Hep-G2 cells (IC_50_ = 14.2, 22.2, and 29.6 μg/mL for *S. album*, *S. austrocaledonicum*, and *S. paniculatum*, respectively).

Sandalwood oil (species not reported) had shown antifungal activity against *C. neoformans* with MIC of 100 μg/mL [26]. Indian sandalwood (*S. album*) had previously shown only marginal activity against *C. albicans* [27] with MIC values of around 600 μg/mL [28,29], consistent with this current investigation. *Santalum album* essential oil had previously demonstrated in vitro cytotoxic activity on both MCF-7 and MDA-MB-231 cells [25,30], as well as several other tumor cell lines [31]. The antifungal and cytotoxic activities of sandalwood oils can be attributed to the high concentrations of α- and β-santalols [32,33].

Both *Cinnamomum cassia* and *C. zeylanica* are rich in cinnamaldehyde (79.9 and 63.9%, respectively), and this compound is likely responsible for the antifungal (MIC = 20, 78, and 78 μg/mL against *C. neoformans*, *A. niger*, and *C. albicans*, respectively) and cytotoxic activities (IC_50_ on MCF-7 = 14.0 and 13.3 μg/mL for *C. cassia* and *C. zeylanicum*, respectively) observed for these essential oils. Both *C. cassia* and *C. zeylanica* have previously shown antifungal activity against *A. niger* [21,34], *C. albicans* [35,36], and *C. neoformans* [37,38], and *C. zeylanicum* has shown cytotoxic activity to MCF-7 and MDA-MB-231 cells [39]. (*E*)-Cinnamaldehyde has been shown to be both antifungal [37,40] and cytotoxic [41].

Aldehydes are major components of the essential oils of cilantro (*Coriandrum sativum* leaf oil, 25.9% (2*E*)-decenal and 7.9% decanal), lemongrass (*Cymbopogon flexuosus*, 49.9% geranial and 23.4% neral), and melissa (*Melissa officinalis*, 30.2% geranial and 23.1% neral). These essential oils showed good antifungal activity against *C. neoformans* (MIC = 20, 78, and 78 μg/mL, respectively) in addition to cytoxicity (IC_50_ ≈ 40, 20–30, and 30 μg/mL, respectively). Citral (a mixture of geranial and neral) has demonstrated both antifungal and cytotoxic activities [13,42,43]. In general, aldehydes are electrophilic agents and can react with nucleophilic biological macromolecules, which may account for the biological activities of aldehydes [44,45,46].

Both patchouli (*Pogostemon cablin*) and vetiver (*Vetiveria zizanoides*) essential oils showed notable antifungal activity against *C. neoformans* (MIC = 20 μg/mL), as well as cytotoxic activity against MCF-7 cells (IC_50_ = 25.0 and 23.9 μg/mL, respectively). Both of these essential oils are rich in sesquiterpene alcohols, patchouli alcohol in *P. cablin*, and (*E*)-isovalencenol and khusimol in *V. zizanoides*. Previous studies on the antifungal activity of patchouli oil showed no activity against *Aspergillus* spp. [21,47], whereas in this work, patchouli oil showed inhibition against *A. niger* with MIC of 156 μg/mL. Likewise, vetiver oil inhibited the growth of *A. niger* and *C. albicans* (MIC = 78 and 313 μg/mL), but previous reports in the literature showed no activity against these two organisms [21,22].

## 4. Materials and Methods

### 4.1. Essential Oils

Commercially available essential oils were obtained from the following sources: dōTERRA International (Pleasant Grove, UT, USA), Améo/Zija International (Lehi, UT, USA), Mountain Rose Herbs (Eugene, OR, USA), and Albert Vielle (Grasse, France). For screening, 1% solutions in dimethylsulfoxide (DMSO) were prepared (i.e., 100 mg essential oil, diluted to 10 g with DMSO).

### 4.2. Gas Chromatography-Mass Spectrometry

Essential oils obtained from dōTERRA International were analyzed by gas chromatography-mass spectrometry (GC-MS) using a Shimadzu GCMS-QP2010 Ultra operated in the electron impact (EI) mode (electron energy = 70 eV), scan range = 40–400 atomic mass units, scan rate = 3.0 scans/s, and GC-MS solution software version. 4.20 (Shimadzu Scientific Instruments, Columbia, MD, USA). The GC column was a ZB-5 fused silica capillary column (Phenomenex, Torrance, CA, USA) with a (5% phenyl)-polymethylsiloxane stationary phase and a film thickness of 0.25 μm. The carrier gas was helium with a column head pressure of 552 kPa and flow rate of 1.37 mL/min. The injector temperature was 250 °C and the ion source temperature was 200 °C. The GC oven temperature program was programmed for 50 °C initial temperature, temperature increased at a rate of 2 °C/min to 260 °C. A 5% *w*/*v* solution of the sample in CH_2_Cl_2_ was prepared and 0.1 μL was injected with a splitting mode (30:1). The remaining essential oils (Ameo, Mountain Rose Herbals, Albert Vielle) were analyzed with an Agilent 6890 GC, Agilent 5973 MSD, EI (70 eV); range of 40–400 amu, scan rate of 3.99 scans/s, HP-5ms column (Agilent Technologies, Santa Clara, CA, USA), He carrier gas, head pressure of 92.4 kPa, flow rate of 1.5 mL/min, GC oven temperature program of 60 °C initial temperature, held for 5 min, then increased at 3 °C/min up to 280 °C, 1% solutions of essential oils in CH_2_Cl_2_, splitless injection. Identification of the oil components was based on their retention indices determined by reference to a homologous series of *n*-alkanes, and by comparison of their mass spectral fragmentation patterns with those reported in the literature [48], and stored in our in-house library [49].

### 4.3. Antifungal Screening

*Candida albicans* (ATCC 18804) and *Cryptococcus neoformans* (ATCC 24607) were grown on potato dextrose agar (PDA) for 48 or 72 h, respectively. Three milliliters of potato dextrose broth (PDB) was inoculated with a single colony. These liquid cultures were grown at 37 °C for another 48 or 72 h for microdilution assays. Minimum inhibitory concentrations (MICs) were determined by microdilution in 96-well round bottom plates from triplicates. Briefly, 100-µL aliquots of MOPS (3-(*N*-morpholino)propanesulfonic acid) buffered RPMI (Roswell Park Memorial Institute) medium pH 7.0 were added to each well. In addition, aliquots of 100 µL of essential oil (1% in DMSO) or the positive and negative controls of 100% DMSO and 100 µM amphotericin B (AMB) and 100% DMSO, respectively, were added to the first row. Each well was serially diluted two-fold down the column excluding the negative control (medium alone). Subsequently, 100 µL of 4 × 10^3^ cells/mL of inoculum in MOPS buffered RPMI were added to each well, resulting in a final concentration of 2 × 10^3^ cells/mL. The microplates were incubated at 37 °C without agitation for 48 or 72 h for *C. albicans* or *C. neoformans*, respectively. MIC values were determined visually as the last well with no turbidity by comparison with the positive and negative controls.

*Aspergillus niger* (ATCC 16888) was grown for seven days at room temperature on potato dextrose agar (PDA) plates. Using an inoculum loop, the spores were gently gathered from the top of the PDA plate and suspended in 1 mL of potato dextrose broth (PDB). Before further use, the spore solution was filtered using sterile cheesecloth. The OD_625_ was adjusted to 0.15 by dilution with fresh PDB. For screening, 100 μL of MOPS buffered RPMI was added to each well of a 96-well plate. A sample or a control of 100 μL was then added to the first well in each row and serially diluted down the column. Lastly, 100 uL of the adjusted spore solution was added to each well of the plate. The plates were incubated at room temperature for seven days. Amphotericin B was used as a positive control, while DMSO and RPMI media alone were used as negative controls. Inhibition was determined visually by comparing the growth of the positive and negative controls with the samples.

### 4.4. Cytotoxicity Screening

Cell culturing and cytotoxicity screening were carried out as previously reported [50]. Briefly, MCF-7 and MDA-MB-231 cells were each grown in sterile RPMI 1640 media with l-glutamine, 26 mL of 7.5% sodium bicarbonate per liter medium; 10,000 units penicillin and 10,000 μg/mL streptomycin per liter of medium; and 15 mL of 1 M HEPES per liter medium, buffered with 5.0 N NaOH to pH 7.35. MCF-7 and MDA-MB-231 cells were plated at concentrations of 1.44 × 10^6^ and 1.44 × 10^4^ cells per well, respectively, in 96-well plates in volumes of 100 μL/well. Test samples were diluted in growth medium to a concentration of 0.01% (*w*/*v*). Tingenone was used as a positive control; growth medium and DMSO were used as negative controls for each plate. The final concentrations of test samples and tingenone controls were 100 μg/mL. The cells were incubated with the test samples at 37 °C and 5% CO_2_ for 48 h. The MTT (3-(4,5-dimethylthiazol-2-yl)-2,5-diphenyltetrazolium bromide) assay was used to determine cell viability. The supernatant medium was removed from each well using suction and a solution of MTT solution (1:10 dilution of 5 mg/mL of stock MTT in growth medium) was added to each well, and the plates were incubated for an additional 4 h at 37 °C and 5% CO_2_. After the incubation period, the medium was carefully aspirated from the wells, and 100 μL of ISO PBS, containing 100 mL isopropyl alcohol, 4.0 μL 5.0 N HCl, and 50.0 mL phosphate-buffered saline, was added to the wells, and the plate was gently shaken to dissolve the crystals. Absorbance was measured using a SpectraMax plate reader at 570 nm and percent viability was determined. For essential oils showing <50% viability, dilutions of the samples (50, 40, 30, 20, and 10 μg/mL) were further assayed. Median inhibitory concentrations (IC_50_) were determined using the Reed-Muench method [51].

### 4.5. Hierarchical Cluster Analysis

The chemical classes of the commercial essential oils, along with the antifungal and cytotoxic activities, were used in the cluster analysis. The 60 essential oils were treated as operational taxonomic units (OTUs) and the 12 chemical classes (monoterpene hydrocarbons, oxygenated monoterpenoids, sesquiterpene hydrocarbons, oxygenated sesquiterpenoids, diterpenoids, aromatic compounds, fatty-acid derivatives, aliphatic esters, aldehydes, phenolics, sulfur-containing compounds, and others) and five bioactivities (*Aspergillus niger*, *Candida albicans*, *Cryptococcus neoformans*, MCF-7, and MDA-MB-231) were used to determine the associations between the essential oils using agglomerative hierarchical cluster (AHC) analysis using XLSTAT Premium, version 19.5.47159 (Addinsoft, Paris, France). Dissimilarity was determined using Euclidean distance, and clustering was defined using Ward’s method.

## 5. Conclusions

Several essential oils have shown notable antifungal activities against opportunistic fungal pathogens. These readily-available materials may add to our treatment options, as agents themselves or as adjuvant therapies, to combat fungal infections. In addition to the antifungal and cytotoxic activities of the essential oils in this study, those essential oil that do not show appreciable cytotoxic activity to human cells may be considered relatively safe for other uses such as cosmetics, flavoring, and aromatherapy.

## Figures and Tables

**Table 1:** Antifungal and cytotoxic activities and major components of sixty commercially-available essential oils. MIC—minimum inhibitory concentrations.

Essential Oil		Source	Antifungal Activity (MIC, μg/mL)			Cytotoxicity (IC_50_, μg/mL, Standard Deviations in Parentheses)			Major Components (>5%)
			*A. niger*	*C. albicans*	*C. neoformans*	MCF-7	MDA-MB-231	Others	
*Abies balsamea*	Balsam fir	Ameo	1250	625	313	50.5 (15.0)	86.7 (7.4)		β-pinene (26.4%), δ-3-carene (18.3%), α-pinene (16.0%), sylvestrene (15.0%), bornyl acetate (9.7%), camphene (5.7%),
*Abies sibirica*	Siberian fir	doTERRA	625	625	156	>100	>100		camphene (24.8%), bornyl acetate (21.1%), α-pinene (15.2%), δ-3-carene (14.6%), limonene (5.7%)
*Anthemis nobilis*	Roman chamomile	doTERRA	625	625	313	>100	>100		α-pinene (15.5%), isobutyl angelate (12.6%), methallyl angelate (10.9%), 3-methylpentyl angelate (5.4%)
*Betula lenta*	Birch	doTERRA	625	625	625	>100	>100		methyl salicylate (99.9%)
*Boswellia carteri*	Frankincense	Ameo	625	1250	313	39.8 (4.1)	50.6 (1.0)		limonene (22.4%), β-caryophyllene (22.2%), *p*-cymene (10.0%), δ-cadinene (9.4%), α-copaene (4.8%)
*Cananga odorata*	Ylang ylang	Ameo	1250	625	78	36.8 (2.3)	61.6 (4.4)	50.4 (7.2) (Hs-578T)	germacrene D (25.0%), β-caryophyllene (15.8%), (*E*,*E*)-α-farnesene (11.0%), benzyl benzoate (8.5%), geranyl acetate (5.2%)
*Cinnamomum cassia*	Cassia	doTERRA	78	78	20	14.0 (1.4)	16.9 (1.0)	16.4 (0.9) (Hep-G2)	(*E*)-cinnamaldehyde (79.9%), (*E*)-*o*-methoxycinnamaldehyde (12.0%)
*Cinnamomum zeylanicum*	Cinnamon	doTERRA	78	78	20	13.3 (1.6)	24.2 (1.5)	25.2 (2.2) (Hep-G2)	(*E*)-cinnamaldehyde (63.9%), eugenol (7.0%), (*E*)-cinnamyl acetate (5.1%)
*Cistus ladanifer*	Cistus	Albert Vielle	625	625	156	36.6 (3.0)	71.1 (5.3)	46.3 (4.0) (Hs-578T)	α-pinene (20.8%), viridiflorene (10.9%),bornyl acetate (6.3%), viridoflorol (5.2%)
*Citrus aurantifolia*	Lime	doTERRA	625	625	313	67.4 (5.9)	40.3 (7.0)		limonene (51.9%), β-pinene (18.8%), γ-terpinene (8.1%)
*Citrus aurantium*	Petitgrain	doTERRA	625	625	313	>100	>100		linalyl acetate (51.5%), linalool (25.4%)
*Citrus bergamia*	Bergamot	Ameo	625	625	313	>100	>100		limonene (34.6%), linalyl acetate (34.3%), linalool (12.7%), γ-terpinene (6.6%), β-pinene (5.6%)
*Citrus limon*	Lemon	doTERRA	625	625	313	94.8 (8.1)	>100		limonene (56.1%), β-pinene (15.8%), γ-terpinene (10.5%)
*Citrus reticulata*	Tangerine	doTERRA	625	625	156	99.8 (10.0)	54.8 (10.7)		limonene (91.3%)
*Citrus sinensis*	Wild orange	doTERRA	625	625	156	87.4 (3.0)	50.4 (11.0)		limonene (94.8%)
*Citrus* × *paradisi*	Grapefruit	doTERRA	313	625	78	79.7 (3.6)	50.6 (8.7)		limonene (91.3%)
*Commiphora myrrha*	Myrrh	Ameo	625	1250	313	>100	86.4 (8.5)		furanoeudesma-1,3-diene (18.1%), curzerene (16.1%), lindestrene (6.9%), α-pinene (6.8%), neryl acetate (6.3%)
*Copaifera officinalis*	Copaiba	Ameo	1250	1250	313	22.7 (1.5)	67.2 (2.2)		β-caryophyllene (87.3%)
*Copaifera* spp.	Copaiba	doTERRA	625	1250	625	60.4 (1.9)	59.8 (6.1)		β-caryophyllene (50.0%), *trans*-α-bergamotene (8.5%), α-copaene (6.8%), α-humulene (6.0%)
*Coriandrum sativum*	Cilantro	doTERRA	313	313	20	42.8 (2.3)	43.1 (3.9)		linalool (29.8%), (2*E*)-decenal (25.9%), (2*E*)-decen-1-ol (10.6%), *n*-decanal (7.9%)
*Coriandrum sativum*	Coriander	doTERRA	625	1250	625	98.6 (4.4)	>100		linalool (73.5%), α-pinene (5.3%)
*Cupressus sempervirens*	Cypress	Ameo	1250	625	313	34.5 (2.6)	65.2 (1.5)		α-pinene (49.7%), δ-3-carene (27.0%)
*Cymbopogon flexuosus*	Lemongrass	doTERRA	313	313	78	23.1 (1.4)	30.7 (2.1)		geranial (49.9%), neral (23.4%), geraniol (7.6%), geranyl acetate (6.4%)
*Elettaria cardamomum*	Cardamom	doTERRA	625	625	156	>100	>100		α-terpinyl acetate (37.2%), 1,8-cineole (35.3%), linalyl acetate (5.0%)
*Eucalyptus radiata*	Eucalyptus	doTERRA	313	625	156	>100	>100		1,8-cineole (78.8%), α-terpineol (8.6%)
*Eugenia caryophyllata*	Clove	doTERRA	156	313	156	>100	>100		eugenol (80.6%), eugenyl acetate (10.5%), β-caryophyllene (6.5%)
*Foeniculum vulgare*	Fennel	doTERRA	625	625	313	95.9 (2.6)	>100		(*E*)-anethole (75.1%), limonene (11.5%), fenchone (6.5%)
*Gualtheria fragrantissima*	Wintergreen	doTERRA	625	625	625	>100	>100		methyl salicylate (99.7%)
*Helichrysum italicum*	Helichrysum	Ameo	1250	625	313	44.8 (1.4)	39.5 (5.7)		neryl acetate (18.3%), α-pinene (18.0%), γ-curcumene (11.6%), β-selinene (10.3%), β-caryophyllene (6.1%), italicene (5.5%), valencene (5.1%)
*Helichrysum italicum*	Helichrysum	doTERRA	625	625	313	81.8 (10.0)	>100		neryl acetate (33.9%), γ-curcumene (14.7%), α-pinene (13.4%)
*Juniperus communis*	Juniper berry	Ameo	625	1250	625	>100	>100		α-pinene (34.9%), myrcene (11.9%), sabinene (11.4%), β-pinene (7.9%), β-caryophyllene (5.1%)
*Juniperus virginiana*	Cedarwood	doTERRA	625	625	313	37.2 (2.2)	35.7 (1.8)		α-cedrene (41.4%), cis-thujopsene (20.0%), cedrol (13.4%), β-cedrene (7.5%)
*Lavandula angustifolia*	Lavender	Ameo	625	625	156	94.7 (4.7)	60.3 (17.3)		linalyl acetate (41.5%), linalool (34.4%)
*Melaleuca alternifolia*	Melaleuca	doTERRA	625	625	625	>100	>100		terpinen-4-ol (47.5%), γ-terpinene (20.2%), α-terpinene (8.6%)
*Melissa officinalis*	Melissa	doTERRA	313	313	78	32.4 (2.5)	28.1 (1.5)		geranial (30.2%), neral (23.1%), β-caryophyllene (13.4%)
*Mentha piperita*	Peppermint	doTERRA	625	625	313	>100	>100		menthol (43.8%), menthone (19.7%), menthyl acetate (6.5%), 1,8-cineole (5.0%)
*Mentha spicata*	Spearmint	doTERRA	313	625	313	>100	>100		carvone (62.3%), limonene (20.1%)
*Myristica fragrans*	Nutmeg	Ameo	625	625	156	43.4 (0.3)	32.6 (1.3)		sabinene (18.8%), myristicin (18.2%), α-pinene (17.1%), β-pinene (11.4%), sylvestrene (5.6%)
*Myrtis communis*	Myrtle	Ameo	1250	313	78	>100	>100		α-pinene (46.1%), 1,8-cineole (27.5%), limonene (9.1%)
*Nardostachys jatamansi*	Spikenard	doTERRA	625	313	156	35.5 (2.2)	65.2 (3.2)		viridiflorene (9.5%), 6,9-guaiadiene (8.8%), valeranone (7.8%), nardosina-7,9,11-triene (6.9%), β-gurjunene (6.7%), valerana-7,11-diene (6.2%), nardol (6.0%)
*Nepeta cataria*	Catnip	Mountain Rose	313	625	156	>100	>100		4aα,7α,7aβ-nepetalactone (58.1%), 4aα,7α,7aα- nepetalactone (20.6%), β-caryophyllene (6.8%)
*Ocimum basilicum*	Basil	doTERRA	313	625	313	>100	>100		linalool (55.7%), 1,8-cineole (9.8%), *trans*-α-bergamotene (5.6%)
*Origanum majorana*	Marjoram	doTERRA	625	625	313	>100	>100		terpinen-4-ol (28.9%), γ-terpinene (14.9%), *trans*-sabinene hydrate (9.5%), α-terpinene (8.7%), sabinene (7.2%)
*Origanum vulgare*	Oregano	doTERRA	156	313	78	35.3 (1.4)	60.1 (17.3)		carvacrol (74.2%), γ-terpinene (5.2%)
*Pelargonium graveolens*	Geranium	Ameo	625	625	625	>100	>100		citronellol (36.6%), *iso*-menthone (5.9%), geraniol (5.5%)
*Picea mariana*	Spruce	Ameo	625	625	313	>100	>100		bornyl acetate (35.9%), camphene (14.5%), α-pinene (14.4%), δ-3-carene (8.2%)
*Piper nigrum*	Black pepper	doTERRA	625	1250	313	87.7 (4.1)	74.0 (3.0)		β-caryophyllene (21.6%), limonene (15.1%), β-pinene (15.1%), sabinene (13.9%), α-pinene (11.1%), δ-3-carene (10.4%)
*Pogostemon cablin*	Patchouli	Ameo	156	625	20	25.0 (5.2)	47.4 (1.1)	22.6 (4.1) (Hep-G2)	patchouli alcohol (36.4%), α-bulnesene (16.3%), α-guaiene (12.4%), seychellene (8.7%), α-patchoulene (5.6%)
*Pseudotsuga menziesii*	Douglas fir	doTERRA	625	313	156	>100	>100		β-pinene (23.0%), sabinene (17.3%), terpinolene (13.5%), δ-3-carene (9.6%), α-pinene (8.1%)
*Rosmarinus officinalis*	Rosemary	doTERRA	625	625	313	>100	>100		1,8-cineole (45.9%), α-pinene (12.0%), camphor (10.9%), β-pinene (6.3%)
*Salvia officinalis*	Sage	Mountain Rose	1250	625	625	>100	>100		*cis*-thujone (27.4%), camphor (21.4%), 1,8-cineole (11.9%), camphene (5.3%), α-pinene (5.2%)
*Salvia sclarea*	Clary sage	Ameo	1250	1250	313	98.4 (3.6)	>100		linalyl acetate (69.0%)
*Santalum album*	Indian sandalwood	doTERRA	313	625	20	9.39 (1.34)	19.3 (0.2)	14.2 (1.6) (Hep-G2)	(*Z*)-α-santalol (45.2%), (*Z*)-β-santalol (25.4%), (*Z*)-α-*trans*-bergamotol (7.8%)
*Santalum austrocaledonicum*	Australian sandalwood	Ameo	313	625	20	9.52 (0.08)	20.4 (1.0)	22.2 (1.4) (Hep-G2)	(*Z*)-α-santalol (49.2%), (*Z*)-β-santalol (23.9%), (*Z*)-lanceol (6.4%)
*Santalum paniculatum*	Hawaiian sandalwood	doTERRA	156	625	20	13.3 (2.4)	23.7 (2.1)	29.6 (1.7) (Hep-G2)	(*Z*)-α-santalol (49.9%), (*Z*)-β-santalol (15.9%), (*Z*)-lanceol (6.6%), (*Z*)-α-*trans*-bergamotol (5.1%)
*Tanacetum annuum*	Blue tansy	doTERRA	625	625	156	>100	>100		sabinene (21.5%), myrcene (14.3%), camphor (12.0%), α-phellandrene (7.4%), *p*-cymene (5.8%), chamazulene (5.0%)
*Thuja plicata*	Arborvitae	doTERRA	313	78	78	89.0 (6.3)	>100		methyl thujate (51.2%), methyl myrtenate (6.6%)
*Thymus vulgaris*	Thyme	doTERRA	156	313	78	>100	>100		thymol (43.9%), carvacrol (14.4%), *p*-cymene (10.5%), β-caryophyllene (7.0%), γ-terpinene (5.1%)
*Vetiveria zizanoides*	Vetiver	doTERRA	78	313	20	23.9 (1.1)	36.2 (0.8)	20.2 (4.4) (Hep-G2)	(*E*)-isovalencenol (13.5%), khusimol (12.1%), α-vetivone (5.4%)
*Zingiber officinale*	Ginger	doTERRA	625	625	313	>100	81.5 (5.9)		α-zingiberene (26.4%), camphene (12.6%), β-sesquiphellandrene (9.2%), *ar*-curcumene (6.5%), β-phellandrene (6.2%), β-bisabolene (5.1%)
